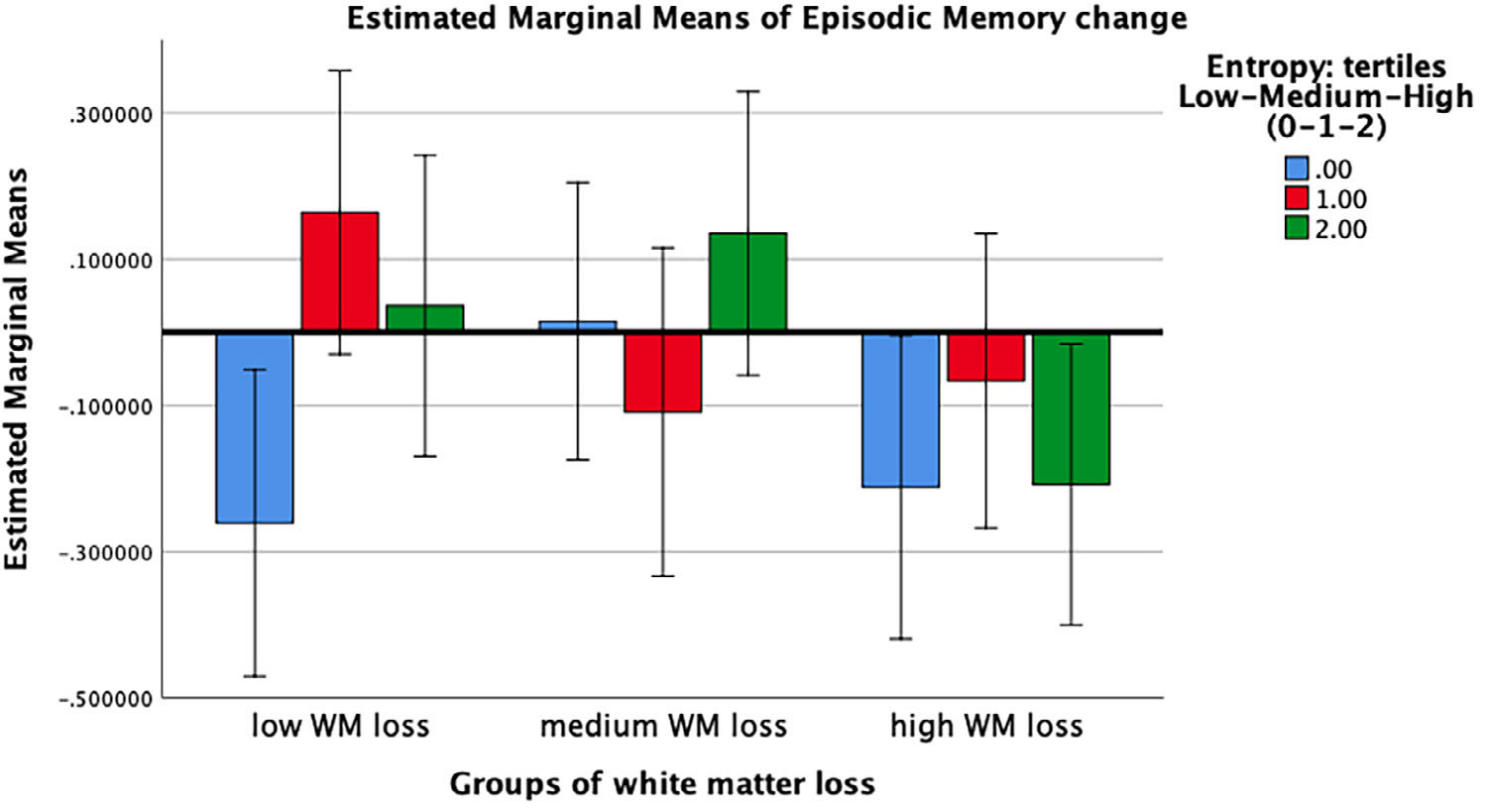# Multilingualism in use and white matter changes amongst healthy middle‐aged and older adults

**DOI:** 10.1002/alz.091444

**Published:** 2025-01-09

**Authors:** Cristina Solé‐Padullés, Gabriele Cattaneo, María Cabello‐Toscano, Lídia Mulet‐Pons, Lídia Vaqué‐Alcázar, Javier Solana Sánchez, Alba Roca‐Ventura, Vanessa Alviarez‐Schulze, Núria Bargalló, Alvaro Pascual‐Leone, David Bartrés‐Faz

**Affiliations:** ^1^ Department of Medicine, Faculty of Medicine and Health Sciences, Institute of Neurosciences, University of Barcelona, Barcelona Spain; ^2^ Universitat Autònoma de Barcelona, Bellaterra (Cerdanyola del Vallès) Spain; ^3^ Guttmann Brain Health Institute, Institut Guttmann, Institut Universitari de Neurorehabilitació Adscrit a la UAB., Badalona, Barcelona Spain; ^4^ Fundació Institut d’Investigació en Ciències de la Salut Germans Trias i Pujol, Badalona, Barcelona Spain; ^5^ Institute of Biomedical Research August Pi i Sunyer (IDIBAPS), Barcelona Spain; ^6^ Sant Pau Memory Unit, Hospital de la Santa Creu i Sant Pau, Biomedical Research Institute Sant Pau, Universitat Autònoma de Barcelona, Barcelona Spain; ^7^ Institut Guttmann, Institut Universitari de Neurorehabilitació adscrit a la Universitat Autònoma de Barcelona, Badalona, Barcelona Spain; ^8^ Fundació Institut d'Investigació en Ciències de la Salut Germans Trias i Pujol, Badalona Spain; ^9^ Institut Guttmann, Institut Universitari de Neurorehabilitació Adscrit a la UAB, Badalona Spain; ^10^ Fundació Institut d’Investigació en Ciències de la Salut Germans Trias i Pujol, Barcelona Spain; ^11^ Universitat Autònoma de Barcelona, Bellaterra Spain; ^12^ Institut Guttmann, Institut Universitari de Neurorehabilitació adscrit a la UAB, Badalona Spain; ^13^ Magnetic Resonance Image Core Facility, Institut d’Investigacions Biomèdiques August Pi i Sunyer (IDIBAPS), Barcelona Spain; ^14^ Centre for Biomedical Research on Mental Health (CIBERSAM), Barcelona Spain; ^15^ Hinda and Arthur Marcus Institute for Aging Research at Hebrew SeniorLife, Boston, MA USA; ^16^ Harvard Medical School, Boston, MA USA; ^17^ Department of Medicine, Faculty of Medicine and Health Sciences, Institute of Neurosciences, University of Barcelona, Barcelona, Spain. Institut d’Investigacions Biomèdiques August Pi i Sunyer (IDIBAPS), Barcelona Spain

## Abstract

**Background:**

Bilingualism can stimulate brain plasticity (Jafari et al. Ann N Y Acad Sci. 2021;1505(1):8‐22) and is also associated with better executive function (Grundy. J Cult Cogn. Sci. 2020;4: 177–199). We investigated whether any feature of multilingualism (age of acquisition (AoA), proficiency or language usage) was related to change in white matter lesions and cognition amongst a cohort of middle‐aged to older adults.

**Method:**

Healthy adults (N=267, age range: 45‐69, 53.56% women) from the bilingual Barcelona Brain Health Initiative cohort (BBHI, Cattaneo et al. Front Aging Neurosci. 2018 11;10) were assembled. Participants completed questionnaires of AoA for a third language (L3), level of proficiency in up to five languages and daily use of languages, up to three languages. Shannon Entropy was computed as an index measuring the proportion of languages used daily in different contexts and participants were classified as monolinguals, bilinguals or multilinguals based on this score. T1 and T2‐weighted acquisitions allowed extraction of volumes of white matter hypointensities (WMH) using Freesurfer at baseline and two years later (N=210). Stepwise regression models were conducted cross‐sectionally to investigate associations between the predicted variables (WMH and cognition) and the following predictors: age, sex, education, AoA, proficiency and Entropy levels. Linear mixed models explored changes in WMH, including fixed effects and a random intercept.

**Result:**

Proficiency (high) and AoA of L3 (after puberty) were retained as factors linked to increased working memory (t=3.49, p=0.001; t=2,88, p=0.004, respectively). High proficiency was also associated with better episodic memory (EM, t=3.19, p=0.002). Multilinguals (high Entropy) presented increased WMH at baseline (t=2.39, p=0.024) and over time (F=4.41, p=0.013). Additionally, there was a slight WMH*Entropy interaction, with a steeper decline in EM amongst monolinguals, regardless of WMH changes, while multilinguals with a medium burden of WMH improved in EM (Figure 1 in green; F=2.39, p=0.065).

**Conclusion:**

Multilingualism in use was linked to increased white matter change and a slightly better maintenance of EM when the burden of structural damage was low to medium. Language usage may provide the brain with an ability to cope with structural brain changes and may be considered a proxy of CR.